# Editorial: Neuromuscular disorders: biomarkers, precision diagnosis, and targeted therapeutics

**DOI:** 10.3389/fnins.2026.1878340

**Published:** 2026-06-11

**Authors:** Marwa Zafarullah, Rupkatha Banerjee, Aditya Singh, Asmita Ghosh, Sandra Almeida

**Affiliations:** 1 Department of Neurology and Neurological Sciences, Stanford School of Medicine, Stanford, CA, United States; 2 Department of Neuroscience, The Herbert Wertheim University of Florida Scripps Institute for Biomedical Innovation and Technology, Jupiter, FL, United States; 3 Department of Neurology, The Lundquist Institute for Biomedical Innovation at Harbor-UCLA, 120 Walter P Martin Research Center, Torrance, CA, United States; 4 Department of Neurosurgery, David Geffen School of Medicine, University of California, Los Angeles, Los Angeles, CA, United States; 5 Department of Genomics and Translational Neuroscience, St. Jude Children's Research Hospital, Memphis, TN, United States; 6 Department of Neurology, University of Massachusetts Chan Medical School, Worcester, MA, United States

**Keywords:** ALS, DM1, glycogenin, lysosomal disorders, myasthenia gravis, Pompe disease, serum creatinine, SMA

Neuromuscular disorders (NMDs) are a heterogeneous group of conditions that impair muscle and/or peripheral nerve function, leading to progressive weakness, sensory deficits, and muscle wasting. Although many NMDs are primarily driven by genetic mutations, their onset and progression are critically influenced by epigenetic mechanisms and environmental interactions, resulting in substantial phenotypic variability and heterogeneous disease trajectories. This genetic and phenotypic complexity underscores the urgent need to identify and develop robust biomarkers that can accelerate the transition from disease onset to molecular diagnosis and inform the assessment of therapeutic efficacy.

The goal of this Research Topic is to showcase studies that not only explore novel biomarkers but also evaluate their utility in clinical settings, with the aim of enabling effective, personalized treatment strategies. By advancing our understanding of the molecular underpinnings of NMDs, this Research Topic seeks to facilitate the translation of biomarker discovery into clinically meaningful, patient-specific therapies. Below, we summarize the nine articles included in this Research Topic, organized by disorder.

Muscular dystrophies (MD) are a subset of NMDs driven by primary genetic defects that lead to progressive muscle degeneration. Despite extensive mechanistic insight, population level frameworks capable of identifying modifiable MD risk factors remain limited. Gong et al. address this gap by examining the association between the Neutrophil Percentage-to-Albumin Ratio (NPAR) and MD prevalence in 3,416 participants. Each unit increase in NPAR was associated with higher odds of MD with a linear dose-response relationship. Importantly, they highlight accelerated biological aging as a potentially modifiable contributor, an aspect largely absent from mutation specific therapies.

Myotonic dystrophy type 1 (DM1) is the most common adult-onset muscular dystrophy, caused by a CTG trinucleotide repeat expansion in the *DMPK* gene that affects muscle, heart, and brain. Zafarullah et al. presented the first exploratory cerebrospinal fluid (CSF) proteomic analysis in DM1, identifying six consistently downregulated candidate biomarkers (CKAP4, SCARF1, NCAM1, CD59, PTH1R, and CA4) and a 15-protein LASSO signature implicating IGF transport, MAPK, and NCAM signaling. Together, these data establish the first CSF proteomic fingerprint for DM1.

Spinal muscular atrophy (SMA) is a progressive autosomal recessive NMD caused by loss-of-function mutations in the *SMN1* gene, leading to degeneration of spinal motor neurons and proximal muscle weakness. Three disease modifying therapies have been approved for SMA, all aimed at increasing SMN protein levels, including nusinersen (an *SMN2* antisense oligonucleotide) and risdiplam, a small molecule *SMN2* splicing modifier. As treatments improve outcomes, reliable biomarkers are essential for monitoring disease course and response. Cheng et al. assessed real world functional biomarker trajectories in patients transitioning from nusinersen to risdiplam across four institutions, demonstrating through motor function score assessments that upper limb motor function was significantly improved from pretreatment baseline at the time of switch, with a sustained upward trend following conversion. Complementing these findings, a retrospective pediatric cohort study by Huang et al. identified serum creatinine as a dynamic biomarker of therapeutic response during nusinersen treatment, outperforming both the creatinine-to-cystatin C ratio and the creatinine muscle index, and supporting its use as a practical, minimally invasive monitoring tool.

Amyotrophic lateral sclerosis (ALS) is a progressive neurodegenerative disease affecting motor neurons, leading to muscle weakness, respiratory failure, and loss of voluntary movement. Its rapid progression emphasizes the critical need to identify genetic triggers and functional biomarkers that enable earlier diagnosis and proactive clinical management. On the genetic front, Shen et al. identified 20 rare *ERBB4* variants in 1,627 Chinese ALS patients, finding that variant carriers experience significantly earlier disease onset. A global meta-analysis confirmed a 0.83% variant frequency, with higher prevalence in Chinese and American populations, positioning *ERBB4* as a prime target for population-specific screening and targeted therapies. A parallel line of inquiry by Rong et al. introduced a noninvasive surface electromyography (sEMG) framework to objectively assess bulbar motor decline in ALS. By detecting neuromuscular irregularities during syllable repetition, the tool accurately distinguished healthy controls from patients with subclinical symptoms, supporting its use for early disease detection and measurement-based care.

Pompe disease (PD), caused by deficiency of lysosomal acid alphaglucosidase (GAA), exemplifies the challenges and successes of enzyme replacement therapy (ERT) in lysosomal disorders. Li et al. reported a 17-year retrospective study of 68 Chinese patients with late onset PD. Mortality was substantially lower among ERT-treated patients than untreated individuals. The predominant GAA variant (W746C) was identified in 43.3% of patients, a population specific diagnostic for East Asian cohorts. Notably, glycogenin accumulation preceded autophagy marker deposition in muscle, raising the hypothesis, that glycogenin may serve as an early pathological biomarker in PD.

Myasthenia gravis (MG) is an autoimmune disorder characterized by fatigable skeletal muscle weakness, primarily driven by pathogenic autoantibodies targeting the neuromuscular junction. MG pathogenesis is increasingly recognized to involve broader immune dysregulation and inflammation beyond autoantibody production, contributing to disease severity and outcomes. Lv et al. reported that serum neurofilament light chain (sNFL) levels are elevated in patients with MG and correlate with disease severity, age, and sex. These findings suggest that sNFL reflects neuromuscular junction damage and secondary neuronal stress, supporting its utility as a biomarker for MG stratification and disease severity. In a second study, Hao et al. reported that the long non-coding RNA FAM225A is significantly reduced in triple-seronegative (triple-SN) MG. FAM225A regulates Th2 differentiation and Th1/Th2 balance and is negatively correlated with disease severity, supporting its potential as a biomarker for stratification and treatment monitoring, as well as a prospective therapeutic target in triple-SN MG.

**Figure 1:**
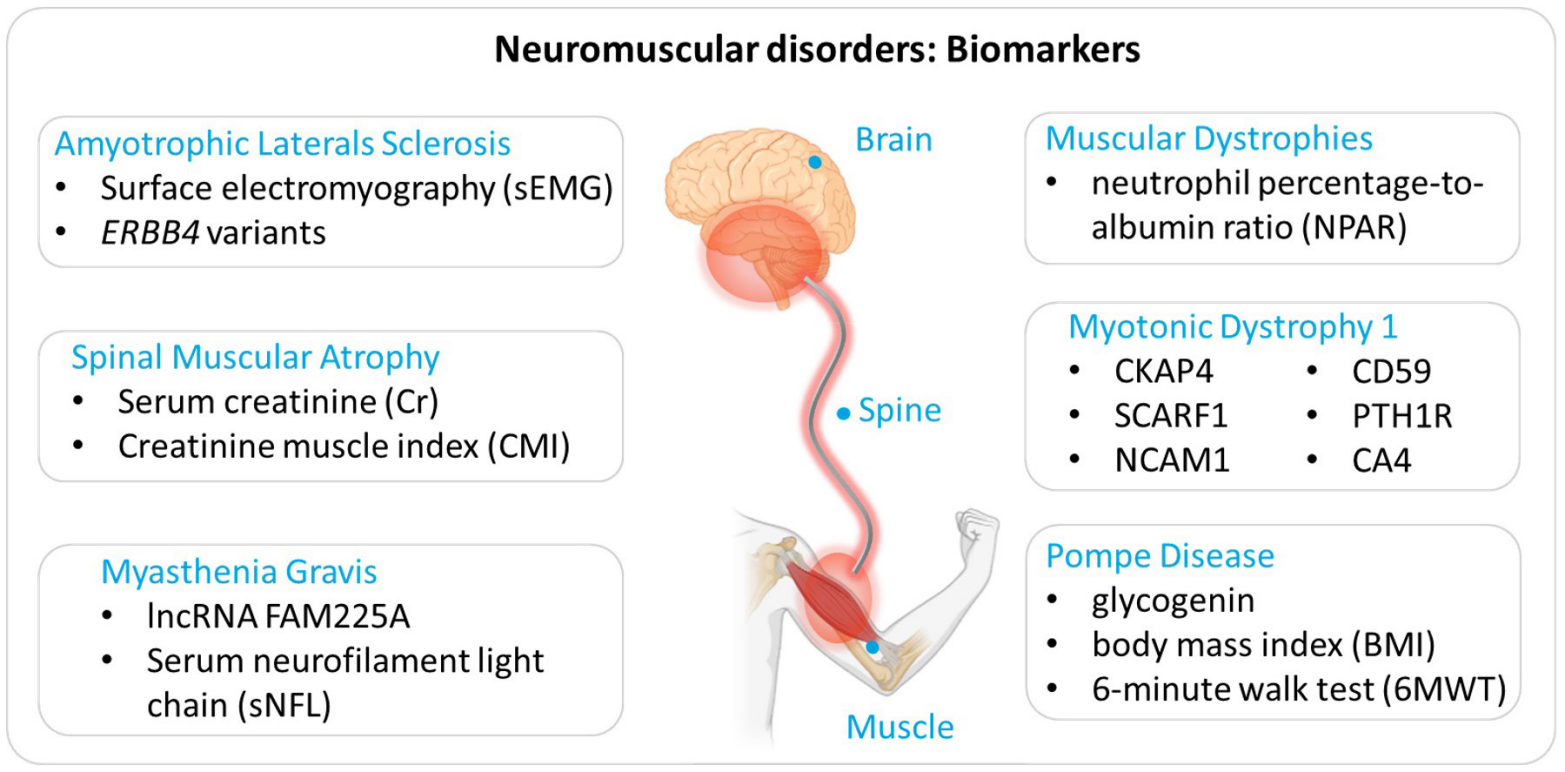
Biomarkers for neuromuscular disorders (NMDs). NMDs represent a diverse group of conditions affecting muscle and/or motor neurons in the brain and spinal cord. This figure summarizes the biomarkers associated with each NMD discussed in this Research Topic. Graphics were created with BioRender.com and MicrosoftPowerPoint.

The articles included in this Research Topic collectively identified and evaluated a broad range of biomarkers for NMDs (Figure 1). While some studies focused on comparing existing biomarkers and diagnostic tests, others introduced and validated novel panels of emerging markers. Collectively, these studies enhance insight into the molecular and clinical complexity of NMDs, contributing to improved diagnostic accuracy and biomarker-based stratification. Overall, this Research Topic advances understanding of NMD pathophysiology, and highlights opportunities to translate biomarker discoveries into personalized diagnostic and therapeutic strategies. Future studies will be essential to validate these findings in larger cohorts and to support their integration into clinical practice.

